# Differential games in the supply chain of innovative products with consumer purchase regret induced returns

**DOI:** 10.1371/journal.pone.0302154

**Published:** 2024-08-05

**Authors:** Xianping Mu, Junjie Liu, Yutao Pu

**Affiliations:** 1 Guizhou Academy of Social Science, Guiyang, China; 2 School of Economics and Management, East China Jiaotong University, Nanchang, China; Federal University of Goias: Universidade Federal de Goias, BRAZIL

## Abstract

Innovative products entering the market will cause dynamic changes in market demand, and consumers’ purchase regret and their return behavior make the market environment more and more complex, which in turn affects the dynamic decision-making in the supply chain. In this paper, under the situation of discrete decision time, combining with the objective reality, we make discrete modification to the classical Bass diffusion model (Bass model), construct a manufacturer-led, retailer-followed supply chain differential game model, analyze the optimal decision-making of the manufacturer and the retailer by combining with the theory of discrete optimal control, and then verify the conclusions by numerical simulation. The results show that: when retailers purchase directly from the manufacturer and sell in the market, the optimal pricing of the innovative product can make the supply chain as a whole, realizing Pareto optimality; consumer’s purchase regret will increase the amount of returns, which will lead to the decrease of product sales and the profits of the manufacturer and the retailer; when the innovative product accounts for a different share of the market, the impact of purchase regret on the wholesale price and the retail price are also different. Therefore, manufacturers need to have an extensive comprehension of the market to minimize the negative effects of consumer regret and returns, and to formulate a reasonable pricing strategy for their products to gain as much profit as possible.

## 1. Introduction

As technology continues to evolve, there are more and more innovative products appearing in the market, such as computers in the 1940s, the invention of commercial cell phones in the 1970s, and the invention of smartphones in the 1990s. The renewal of innovative products is conducive to meeting the diversified needs of people’s lives, and greatly improving the quality of life. The entire process from raw material in the hands of the manufacturer to finished product in the hands of the consumers is a kind of mesh chain structure, the supply chain is such a chain connecting the suppliers and customers [1]. And through this mesh chain, the raw materials will be transformed into products by the enterprise, and ultimately sold to the customers, to obtain economic benefits [2].

The firm’s economic benefits often depend on the decision-making strategies of the decision makers in the supply chain. However, the formulation of strategies is accompanied by a variety of factors, such as the uncertainty of demand caused by the entry of innovative products into the market [3], the uncertainty of consumer behavior [4], the uncertainty of the market environment [5], the uncertainty of the government’s policies [6] and so on. These uncertainties affect the rational pricing of products, which in turn affects product sales and further has an impact on manufacturers’ profits. Among these uncertainties, uncertainty in demand and consumer behavior tends to have a more direct impact on the decision making of decision makers in the supply chain. For example, in March 2020, Huawei’s cell phone sales reached 38.5% in Jindong’s own sales, and in May of the same year, sales of Huawei’s cell phones were dealt a fatal blow due to sanctions, and in December of the same year, Huawei’s cell phone sales were only 7.9%. However, on August 29, 2023, after the release of Huawei Mate60 series, there was a situation of "hard to find" in the market. According to Counterpoint, Huawei’s sales grew by 37% in Q3 2023, while Apple’s sales fell by 10% year-on-year. Obviously, the demand for products from consumers in the market is dynamic, and when an innovative product enters the market, its reputation will be affected by advertising, consumer feedback, and media evaluation, etc., and consumer behavior and psychology will change, thus affecting the optimal decision-making of each node manufacturer [7].

The theory of discrete-time optimal decision-making holds that affected by the lag of market feedback, the decision maker needs to make decision-making adjustments based on market feedback, so the decision result obtained is not a continuous decision path decisive path but a discrete decisive sequence. For example, on October 18, 2023, the price of iPhone 14 128G and 256G versions were reduced by CNY600 and 512G version by CNY300; the price of iPhone 14 Plus 128G version was reduced by CNY1000, 256G version was reduced by CNY900, and 512G version was reduced by CNY700, and the change of the product price did not occur every day. Wei et al. [8] pointed out that the optimal decision obtained under continuous time is an ideal state in theory, while the optimal decision obtained under discrete time is a realistic norm. Unfortunately, the importance of discrete-time optimal decision-making has not been emphasized from the research background, especially the impact of consumer behavior and product demand on optimal pricing, product sales, and manufacturer’s optimal profit in the discrete-time scenario, which has not been addressed in the existing literature.

In order to illustrate the impact of consumer behavior and innovative product demand on the optimal decision making in the discrete time supply chain, this study focuses on a typical innovative product supply chain model, i.e., there exists only one dominant manufacturer and one follower retailer in the market, who play a non-cooperative differential game among them. The entry of innovative products into the market causes dynamic changes in market demand, which leads to difficulties in describing the product demand function. In order to solve this problem, scholars have proposed the concept of innovative product diffusion model (Bass model). In this paper, we use the Bass model to explore the impact of consumers’ purchase regret and its induced return behavior on the optimal decisions of decision makers in the supply chain as well as on the profits of manufacturers in a discrete-time situation.

In order to explore the impact of consumer’s purchase regret and its induced return behavior on the optimal strategies and profits of supply chain members in discrete-time situations, this paper, based on the Bass model used in reference [7], further introduces the consumer’s purchase regret and its induced return behavior into the Bass model, and discretizes the Bass model and constructs a supply chain differential game model with the manufacturer as the dominant one and the retailer as the follower one, to explore the impact of the consumer’s purchase regret and its induced return behavior on the optimal strategies of the supply chain members after the innovative product enters into the market.

This paper focus on the following questions:

1. How do product prices change during the sales process as decision-makers in the supply chain price products more realistically?
2. As the sales process progresses, the innovative product spreads through the market, how does the change in the share of the innovation in the market affect the pricing of the product?
3. Consumers in the market will develop purchase regret, which will further lead to consumer return behavior. What is the effect of the consumer purchase regret and the ensuing return behavior on supply chain decision-makers’ profitability and capacity to make the best decisions?

The structure of this paper is organized as follows: Section 2 reviews the existing studies. Section 3 presents the modeling assumptions of the paper. Section 4 is the modeling and solving. Numerical analysis is presented in Section 5. In Section 6 the conclusion of the paper is obtained.

## 2. Literature review

In this section, we review the research related to optimal decision-making in supply chains, innovative product diffusion models, and consumer regret psychology, and discuss supply chains under discrete time. This section serves as the fundamental basis for the study conducted in this publication.

### 2.1. Optimal decision of supply chain

Existing studies related to supply chains can be categorized into supply chains under static time and supply chains under dynamic time. Supply chains under static time assume that the decision makers’ decisions do not change over time, and once a decision is made, it is stationary. Macchion et al. [9] addressed the tension between supply chain partners introduced by a sustainability program by considering the static complexity of the supply chain. Wu et al. [10] examined a supply chain involving a cautious retailer and a supplier that offers a loss-sharing and trade credit, and discovered that by enhancing trade credit and sharing losses, the supplier can enhance both the store’s satisfaction and its own profitability. Gu et al. [11] studied strategic sourcing and supplier financing strategies for downstream manufacturers with peer and synergistic competition to provide optimal decision-making strategies for manufacturers and suppliers. Li et al. [12] examined rural household incomes affected by the epidemic by constructing an OLS linear regression model of a supply chain with logistical disruptions. Zahra et al. [13] investigated the impact of the bargaining phenomenon on the level of product greening and the overall profitability of the supply chain in both centralized and decentralized models. However, decision-making is influenced by multiple factors, and in real life, the conditions of these factors affecting decision-making will change over time, and research on supply chains in static time is flawed.

Some scholars have also argued that in a dynamic time scenario, the decisions made by the members of the supply chain are always changing, and the adjustments in the decision maker’s strategy occur continually over time, so these scholars hypothesize that the formulation of the decision maker’s strategy in the supply chain is related to the change in time, and the supply chain under dynamic time is investigated. Ni et al. [14], by investigating process innovation and contractual decisions in supply chains, found that manufacturers with efficient innovation are subject to a double marginalization effect and higher wholesale prices due to longer contract durations. Alireza et al. [15] studied the recovery problem of a dynamic supply chain by constructing a new generalized model based on bounded optimal control theory to respond to the type, extent, and timing of measures taken by the supply chain. Liu et al. [7] used the diffusion of innovation model to construct a supply chain model for remanufacturing innovative products recovered by retailers, and used optimal control theory to analyze the impact of innovative products entering the market on the optimal decisions of each node enterprise. Wei et al. [8] examined the effects of sharing real-time demand information on the development of environmentally friendly technology and financial gains in a supply chain by constructing a supply chain consisting of one manufacturer and one retailer. Huang [16] considered three scenarios, namely, absence of government involvement, government intervention, and collaboration facilitated by government intervention, and investigated the operation of an urban-rural dual-market low-carbon supply chain consisting of a single manufacturer and two retailers.

### 2.2. Supply chain in discrete time

In the real situation, the change of strategy is not continuous, each strategy needs to be implemented in the supply chain for a period of time. Only when the elemental conditions affecting the decision change, the decision maker will formulate a new strategy again at the next point in time, and the decision maker will combine the optimal strategies at each point in time to finally obtain a discrete optimal decision sequence. For example, after the new energy car Tesla is listed, the manufacturer adjusts the price according to the market feedback and policy changes, for example, the price of Model Y high-performance version increased by 0.2 million RMB on February 17, 2023, the price increased 0.2 million RMB on May 2, and the price decreased 1.4 million RMB on August 14, 2023. Compared with the continuous-time dynamic scenario, the discrete time dynamic scenario is more in line with the real situation, and the study of the supply chain under the discrete time dynamic scenario is more realistic and practical. Kastsian et al. [17] proposed a method for general nonlinear discrete time based on the method of normal vectors for dynamic systems with uncertain parameters and applied the method to discrete time supply chain modeling for research. Wei et al. [18] studied a supply chain inventory management system consisting of a single manufacturer and a single retailer in a discrete time scenario and found that the fluctuation of the retailer’s inventory production is independent of the manufacturer’s delivery frequency. Antonio et al. [19] studied the capacity constraint problem in a two-tier supply chain and reduced the impact of changeover time from one product to another on supply chain performance by developing a production planning strategy. Liu et al [20] constructed a dynamic green competition model that incorporates finite rationality and utilizes a gradient adjustment mechanism to analyze a green supply chain comprising a single supplier and two manufacturers.

### 2.3. Innovation product diffusion model

In order to be more competitive in the market, manufacturers need to innovate their products and the introduction of the product innovation can have an impact on supply chain decisions. Unlike general products, innovative products refer to the research and development of new products as well as the upgrading of old products [21]. Golrizgashti et al. [22] studied the introduction of product innovation into sustainable supply chains and realized an increase in manufacturers’ market competitiveness. Li et al. [23] investigated product innovation and decision-making strategies under different supply chain contract models. Most of the existing studies only considered the companies’ focus on product innovation. Innovative products, such as innovative sweeping robots and newly released smartphones, are new and unfamiliar to the market as well as to consumers. In order to predict the sales of innovative consumer durables in the market, Bass proposed an innovative product diffusion model (Bass model) [24]. Robinson et al. [25] studied the model in more depth by considering the effect of the price factor. Cesare et al. [26] used the differential game theory to obtain the optimal product price and advertising strategy. It has also been argued that the discrete Bass model gives simpler significantly superior outcomes compared to the continuous Bass model [27].

### 2.4. Consumers’ purchase regret psychology

After purchasing and trying a product, consumers may think that the product does not meet their expectations and it is regretful to purchase the product. This regret psychology causes consumers to advertise negatively about the product they have purchased, which can damage the goodwill of the product [28]. Huang et al. [29] from the perspective of gender differences between men and women, explored the different mentalities that consumer gender differences exhibit when making choices. Jian et al. [30] investigated the impact of expected regret from discounted products on supply chain decision-making and coordination under static time and designed a gain-sharing contract to optimise the profits of the supply chain and its members. Liu et al. [31] constructed a manufacturer-driven green supply chain game model to explore the impact of consumers’ expected regret on the optimal pricing and greening level of manufactured products. Some scholars have also further analysed the impact of regret-induced return behaviour on the supply chain. By constructing a model of retailer pricing and inventory optimisation. Shi et al. [32] investigated the issue of pre-sale strategy for retailers by considering consumer pre-purchase regret behaviour. Sergio et al. [33] examined the effect of mindstream awareness on process regret and outcome regret, and further explored the impact of purchase regret on product return mechanisms in the context of e-commerce retailers.

### 2.5. Research gap

By combing through the existing studies, we find that the research on dynamic decision-making in supply chain is multi-faceted, among which static decision-making and dynamic decision-making in continuous time are the most common. However, few scholars have studied dynamic decision-making in discrete time. Reference [10] has studied the optimal decision-making problem of supply chains only in the static case. Although reference [7] investigated the dynamic optimization problem of a supply chain using the Bass model, the decision time was assumed to be a continuous ideal rather than a discrete constant as studied in this paper. Reference [19, 20] explored the optimal decision making problem of supply chain in discrete time situation, but the study did not consider the impact caused by the innovative products entering the market, reference [26, 27] studied the optimal decision making problem of supply chain by using the Bass model, but did not consider the impact of the consumers’ purchase regret psychology on the optimal decision making of the manufacturers, the study of the reference [31, 34] showed that that consumer purchase regret and its derivative behaviors affect the decision makers’ decision making, which further affects the supply chain and the profits of the chain members. In this paper, we use differential game theory to study the optimal decision-making of supply chain decision-makers in discrete time by considering the diffusion of innovative products into the market, and further explore the impact of consumers’ purchase regret and their return behavior on the optimal pricing of the products, product sales, and profits of the manufacturers, etc., and finally use numerical simulation to verify the reliability of the conclusions obtained.

This paper is a more in-depth study of existing research: (1) The majority of studies on supply chain focus on static time periods [10], and some scholars also study under continuous dynamic time [7]. Based on this, this paper provides some theoretical foundation for the later dynamic study of supply chain with discrete decision time. Specifically, this paper extends the use of the Bass model by discretizing the Bass model to study the vendors’ pricing at various stage. To the best of the authors’ knowledge, this is the first time in the reference that the optimal dynamic decision-making problem of a supply chain has been investigated using differential game theory in a discrete decision-time scenario. (2) This paper introduces consumer purchase regret into the supply chain. In contrast to studies that also consider the psychology of consumer behavior and return behavior [34], this study replaces the psychology of consumer anticipation with the psychology of purchase regret, and correlates the psychology of consumer regret with return behavior in discrete time.

Table 1 explains the gaps that exist between this paper and existing research.

**Table 1:** Comparison between previous reference and this paper.

Author(s)	Optimal decision of supply chain	Supply chain in discrete time	Bass model	Consumers ’ purchase regret psychology
Liu et al. [7]	√	—	√	—
Wu et al. [10]	√	—	—	—
Antonio et al.[19]	√	√	—	—
Liu et al.[20]	√	√	—	—
Cesare et al. [26]	√	—	√	—
Satoh [27]	—	—	√	—
Liu et al. [31]	√	—	—	√
Chen et al.[34]	√	—	—	√
This study	√	√	√	√

The main innovations of this paper are: (1) Considering the price effect, the consumer purchase regret psychology, its caused return behavior and other factors into the classical Bass model, then discrete processing the Bass model, a sales volume function that is more relevant to the reality than the continuous one is obtained. (2) Under the situation of discrete decision time, a differential game model is constructed to analyze the impact of consumers’ purchase regret psychology and its caused return behavior on product sales, optimal pricing, and optimal profit of each manufacturer in the supply chain, which further enriches the theory of consumer behavior, the theory of supply chain management and their combination.

## 3. Model assumptions

The research in this paper focuses on a supply chain that comprises only one manufacturer and one retailer. The manufacturer, who is responsible for manufacturing and wholesaling innovative products, is the dominant player in the supply chain; the retailer, who is responsible for selling innovative products, is the follower of the supply chain, with full transparency of information about each other.

The symbols and meanings used in this paper are shown in Table 2.

**Table 2:** Symbols and meanings used in the supply chain.

Symbols	Meanings
*c*_*m*_	Manufacturer’s marginal unit cost of producing an innovative product.
*c*_*r*_	The retailer’s marginal unit cost for ancillary purchases (e.g., transport, storage, etc.).
*w*_*i*_	The wholesale price at which the manufacturer sells the innovative product in the stage *i*, as a control variable for the manufacturer.
*p*_*i*_	The retail price at which the retailer sells the innovative product in the stage *i*, as a control variable for the retailer.
*x*_*i*_	The number of people buying innovative products in the consumer market in the stage *i*.
*m*_*e*_	Innovative products return rate.
*a*	The effect of consumer regret on return behavior.
*ζ*	The regret coefficient of consumers after purchasing the product.
*ρ*	The price elasticity of demand for the product.
*n*	The quantity of stages of decision-making by members of the supply chain.
*N*	The aggregate quantity of prospective consumers in the market.
*D*_*i*+1_	The actual volume of innovative products sold during stage *i* + 1.

In order to make the model in this paper more in line with the real situation and to make it clear, the following assumptions are made about the model:

**H1**: According to Liu et al. [7], this paper assumes that the manufacturer’s marginal unit cost of producing the innovation is *c*_*m*_, the marginal unit cost that the retailer spends on ancillary purchases (e. g. transport, storage, etc.) is *c*_*r*_, and there is *c* = *c*_*m*_ + *c*_*r*_. In a discrete-time supply chain, the decisions of the participants in the supply chain change with the stage of decision-making, the paper assumes that the number of stages is *n*. In stage *i* + 1, the manufacturer’s wholesale price of the innovative product is *w*_*i*_, and the retailer’s retail price of the innovative product is *p*_*i*_. Thus, the manufacturer’s sales profit on the sale of one innovative product is *w*_*i*_ − *c*_*m*_, the retailer’s sales profit on the sale of one unit of the innovative product is *p*_*i*_ − *w*_*i*_ − *c*_*r*_; and the potential total number of consumers in the market is *N*.

Bass, an American management psychologist, pointed out that the diffusion process of an innovative product entering the market is similar to the spreading process of a virus in the crowd: when a pathogen spreads in the crowd, it not only infects the crowd, but also spreads in the crowd, and at the same time, susceptible people in the crowd will be infected by the influence of the infected crowd; this is similar to the situation when an innovative product enters the market, and the virulence of the virus can be regarded as the virulence of the virus can be regarded as the attraction of the innovative product to the consumers in the market, while the infected and susceptible groups correspond to the innovators and imitators in the consumer market respectively. The purchasing behavior of innovators is only related to external factors such as advertising, and its proportion is *F*(*t*). Their purchasing decisions are independent of other consumers in the market; the purchasing behavior of imitators is related to internal factors such as comments and feedbacks from other consumers, and its proportion is 1 − *F*(*t*). Their purchasing decisions are influenced by the market, and this influence increases along with the increase in the number of purchasers, which is called the diffusion model of innovative products. Bass called the innovative product diffusion model (Bass model) [24]. Many research have shown that the Bass diffusion model is one of the main analytical tools used by the market to forecast the demand for new products and technologies [25–27], therefore, this paper uses the Bass model as a base model to study the supply chain of an innovative product with returns caused by consumer regret, the Bass model can be used to more graphically depict the diffusion process of an innovative product after it enters the supply chain. The differential equation of the Bass model is expressed in the form:

$$\frac{\text{d}F(t)}{\text{d}t}=(p+qF(t))(1-F(t))$$ (1)

Combining the Bass model in continuous time, which takes into account the price factor [25], with the discrete-time Bass model [27], the incremental number of purchasers in the stage *i* + 1 is obtained as *x*_*i*+1_ − *x*_*i*_ = (*j* + *kx*_*i*_)(1 − *x*_*i*_), where *j* denotes the coefficient of external influence, *k* denotes the parameter of internal influence; *j*(1 − *x*_*i*_) denotes the proportion of adoption that purchased the innovation due to external influence, namely the proportion of innovative consumers, and *kx*_*i*_(1 − *x*_*i*_) denotes the proportion of adoption that purchased the innovation due to the influence of previous purchasers, namely the proportion of imitative consumers.

This paper also hypothesizes that there are two influencing factors in the process of purchasing the product:

**H2**: After the use of the product, because the product does not meet their own psychological expectations, quality is not against the price and other reasons, consumers will develop a sense of regret for purchasing this product, called consumer purchase regret psychology [28]. Therefore, according to Davvetas et al. [28], these dissatisfied customers will boycott the product, leading to a substantial decline in the product’s reputation. Consequently, this will greatly impede the spread of the new product in the market. Assuming that the proportion of consumers with regret in the stage *i* is *ζx*_*i*_.
**H3**: Based on Robinson et al. [25], this paper assumes that the higher the price of the product, the weaker the consumer’s desire to buy, so the price will also have an impact on the diffusion of innovative products.

Thus, the Bass diffusion model with consumer purchase regret in the discrete time case is obtained as:

$$x_{i+1}-x_{i}=e^{-\rho p_{i}}(j+k(1-\zeta )x_{i})(1-x_{i})$$ (2)

Where *ζ* is the coefficient of regret of consumers who regret purchasing the innovative product and advertise the product negatively, hampering the diffusion of the innovative product; *k* is the imitation coefficient, where potential consumers in the market are influenced by the favorable reputation of the goods and engage in mimicry buying; *ρ* is the coefficient of consumer sensitivity to the price of the product; $e^{-\rho p_{i}}$ indicates the impact of changes in product prices on the sales of innovative products, with a gradual increase in product prices, a gradual low demand for the product and a decrease in sales. Therefore, the market demand for the innovative product in the stage *i* + 1 can be expressed as $D_{i+1}^{\prime }=N(x_{i+1}-x_{i})$.

**H4**: Consumer purchase regret psychology also prompts their return behavior, which has an impact on product sales, the return odds of consumers is positively correlated with their own purchase regret psychology. Therefore, the innovative product return rate can be expressed as follows: *m*_*e*_ = *aζ*, where *a* denotes the effect of consumer regret on returns [34]. Therefore, according to Chen et al. [34], this paper assumes that the modified Bass model representation is obtained as: $x_{i+1}-x_{i}=(1-a\zeta )e^{-\rho p_{i}}(j+k(1-\zeta )x_{i})(1-x_{i})$. This model captures the impact of consumer purchase regret and the resulting return behavior on the share of innovative products in the market., the actual sales volume of the innovative products in the stage *i* + 1 is $D_{i+1}=Ne^{-\rho p_{i}}(1-a\zeta )(j+k(1-\zeta )x_{i})(1-x_{i})$. The manufacturer’s sales revenue in the stage *i* + 1 is *w*_*i*_*D*_*i*+1_, and its cost of selling is *c*_*m*_*D*_*i*+1_, and the retailer’s sales revenue in the stage *i* + 1 is *p*_*i*_*D*_*i*+1_, and its cost of selling is (*w*_*i*_ + *c*_*r*_)*D*_*i*+1_. Thus, the manufacturer’s profit on sales in the stage *i* + 1 is $\pi_{M_{i+1}}=(w_{i}-c_{m})D_{i+1}$, and the retailer’s profit on sales in the stage *i* + 1 is $\pi_{R_{i+1}}=(p_{i}-w_{i}-c_{r})D_{i+1}$.

## 4. Model building and solving

In this discrete-time differential game model of the supply chain, the dominant manufacturer first determines its wholesale price *w*_*i*_ for the innovative product, followed by the follower retailer who determines its retail price *p*_*i*_. The profit functions of the manufacturer and the retailer are respectively:

$$\pi_{M}=\sum_{i=0}^{n-1}(w_{i}-c_{m})D_{i+1}$$ (3)

$$\pi_{R}=\sum_{i=0}^{n-1}(p_{i}-w_{i}-c_{r})D_{i+1}$$ (4)

The respective objective functions of the manufacturer and the retailer are thus obtained as:

$$max\left\{\pi_{M}=\sum_{i=0}^{n-1}\left(w_{i}-c_{m})e^{-\rho p_{i}}N(1-a\zeta )(j+k(1-\zeta )x_{i})(1-x_{i}\right)\right\}$$ (5)

$$max\{\pi_{R}=\left\{\sum_{i=0}^{n-1}\left(p_{i}-w_{i}-c_{r})e^{-\rho p_{i}}N(1-a\zeta )(j+k(1-\zeta )x_{i})(1-x_{i}\right)\right\}$$ (6)

$$s.t.\left\{\begin{array}{l} x_{i+1}-x_{i}=e^{-\rho p_{i}}(j+k(1-\zeta )x_{i})(1-x_{i})\\ x_{0}=0 \end{array}\right.$$ (7)

**Proposition 1**: The optimal sequence of manufacturers’ wholesale prices {*w*_*i*_} is:

$$w_{i}=\frac{1}{\rho }-\frac{\lambda_{i+1}}{N(1-a\zeta )}+c_{m}$$ (8)

The optimal sequence of retailer’s retail price {*p*_*i*_} is:

$$p_{i}=\frac{1}{\rho }-\frac{\lambda_{i+1}}{N(1-a\zeta )}+w_{i}+c_{r}$$ (9)

And there is a transverse condition: $\lambda_{i}=\frac{N(1-a\zeta )}{\rho }e^{-2+\frac{2\rho }{N(1-a\zeta )}\lambda_{i+1}-c\rho }(k(1-2x_{i})(1-\zeta )-j)$, *λ*_*n*_ = 0.

**Proof**: According to the order of the difference game, the optimal sequence {*p*_*i*_} of retailer’s retail price is calculated first.

The Hamilton function of the retailer is obtained from the discrete optimal control theory as:

$$H_{R_{i}}=((p_{i}-w_{i}-c_{r})N(1-a\zeta )+\lambda_{R_{i+1}})e^{-\rho p_{i}}(j+k(1-\zeta )x_{i})(1-x_{i})$$ (10)

{*p*_*i*_} is obtained by solving the necessary condition $\frac{\partial H_{R_{i}}}{\partial p_{i}}=0$:

$$p_{i}=\frac{1}{\rho }-\frac{\lambda_{R_{i+1}}}{N(1-a\zeta )}+w_{i}+c_{r}$$ (11)

Joining Eqs (10) and (11) and the transverse conditional equation $\lambda_{R_{i}}=\frac{\partial H_{R_{i}}}{\partial x_{i}}$, the solution yields that:

$$\begin{array}{l} \lambda_{R_{i}}=\frac{\partial H_{R_{i}}}{\partial x_{i}}=\frac{N(1-a\zeta )}{\rho }e^{-1+\frac{\rho }{N(1-a\zeta )}\lambda_{R_{i+1}}-(w_{i}+c_{r})\rho }\\ \left(k(1-2x_{i})(1-\zeta )-j\right) \end{array}$$ (12)

and a terminal constraint $\lambda_{R_{n}}=0$.

The optimal sequence of the manufacturer’s wholesale prices $\left\{w_{i}\right\}$ is then calculated. Combined with Eq (11), the manufacturer’s Hamilton function is:

$$\begin{array}{l} H_{M_{i}}=((w_{i}-c_{m})N(1-a\zeta )+\lambda_{M_{i+1}})e^{-1+\frac{\rho }{N(1-a\zeta )}\lambda_{R_{i+1}}-\rho (w_{i}+c_{r})}\\ \left(j+k(1-\zeta )x_{i})(1-x_{i}\right) \end{array}$$ (13)

And {*w*_*i*_} can be obtained from the necessary condition $\frac{\partial H_{M_{i}}}{\partial w_{i}}=0$:

$$w_{i}=\frac{1}{\rho }-\frac{\lambda_{M_{i+1}}}{N(1-a\zeta )}+c_{m}$$ (14)

Joining Eqs (13) and (14) and the transverse conditional equation $\lambda_{M_{i}}=\frac{\partial H_{M_{i}}}{\partial x_{i}}$, the solution yields that:

$$\begin{array}{l} \lambda_{M_{i}}=\frac{\partial H_{M_{i}}}{\partial x_{i}}=\frac{N(1-a\zeta )}{\rho }e^{-2+\frac{\rho }{N(1-a\zeta )}(\lambda_{R_{i+1}}+\lambda_{M_{i+1}})-c\rho }\\ \left(k(1-2x_{i})(1-\zeta )-j\right) \end{array}$$ (15)

And a terminal constraint $\lambda_{M_{n}}=0$.

Substituting Eqs (14) into (12) reveals that $\lambda_{R_{i}}=\lambda_{M_{i}}$. And when $\lambda_{i}=\lambda_{R_{i}}=\lambda_{M_{i}}$, it gives that $w_{i}=\frac{1}{\rho }-\frac{\lambda_{i+1}}{N(1-a\zeta )}+c_{m}$, $p_{i}=\frac{1}{\rho }-\frac{\lambda_{i+1}}{N(1-a\zeta )}+w_{i}+c_{r}$,$\lambda_{i}=\frac{N(1-a\zeta )}{\rho }e^{-2+\frac{2\rho }{N(1-a\zeta )}\lambda_{i+1}-c\rho }(k(1-2x_{i})(1-\zeta )-j)$, and *λ*_*n*_ = 0.

**Proposition 2**: The incremental share of the innovative product in the market in the stage *i* + 1 is:

$$x_{i+1}-x_{i}=e^{-2+\frac{2\rho }{N(1-a\zeta )}\lambda_{i+1}-c\rho }(j+k(1-\zeta )x_{i})(1-x_{i})$$ (16)

**Proof**: From Proposition 1, it can be calculated that $p_{i}=\frac{1}{\rho }-\frac{\lambda_{i+1}}{N(1-a\zeta )}+w_{i}+c_{r}$, $w_{i}=\frac{1}{\rho }-\frac{\lambda_{i+1}}{N(1-a\zeta )}+c_{m}$. And from Eq (7), it can be calculated that: $x_{i+1}-x_{i}=e^{-2+\frac{\rho }{N(1-a\zeta )}(\lambda_{R_{i+1}}+\lambda_{M_{i+1}})-c\rho }(j+k(1-\zeta )x_{i})(1-x_{i})$, where $\lambda_{i}=\lambda_{R_{i}}=\lambda_{M_{i}}$. Therefore, $x_{i+1}-x_{i}=e^{-2+\frac{2\rho }{N(1-a\zeta )}\lambda_{i+1}-c\rho }(j+k(1-\zeta )x_{i})(1-x_{i})$.

**Corollary 1**: The wholesale prices {*w*_*i*_} and the retail prices {*p*_*i*_} of an innovative product are both related to the cross-sectional condition *λ*_*i*+1_. As *λ*_*i*+1_ decreases, both {*w*_*i*_} and {*p*_*i*_} increase, and 2*w*_*i*_ − *p*_*i*_ = *c*_*m*_ − *c*_*r*_.

**Proof**: From Proposition 1, $\lambda_{i}=\frac{N(1-a\zeta )}{\rho }e^{-2+\frac{2\rho }{N(1-a\zeta )}\lambda_{i+1}-c\rho }(k(1-2x_{i})(1-\zeta )-j)$, where 1 − *aζ* > 0. Because *j* > *k*, *j* > *k*(1 − 2*x*_*i*_)(1 − *ζ*), *λ*_*i*_ < 0. *λ*_*i*_ decreases as the number of stages increases. The partial derivatives of first orders of *λ*_*i*+1_ for *w*_*i*_ and *p*_*i*_, respectively, yields $\frac{\partial w_{i}}{\partial \lambda_{i+1}}=-\frac{1}{N(1-a\zeta )}<0$, $\frac{\partial p_{i}}{\partial \lambda_{i+1}}=-\frac{2}{N(1-a\zeta )}<0$. Therefore, *w*_*i*_ and *p*_*i*_ are negatively correlated with *λ*_*i*+1_, with *w*_*i*_ and *p*_*i*_ increasing throughout the sales process. Substituting *w*_*i*_ into *p*_*i*_ and solving yields $p_{i}=\frac{2}{\rho }-\frac{2\lambda_{i+1}}{N(1-a\zeta )}+c_{m}+c_{r}$, which gives 2*w*_*i*_ − *p*_*i*_ = *c*_*m*_ − *c*_*r*_.

**Corollary 2**: When the proportion of innovative products in the market is below $\frac{1}{2}+\frac{aj\rho \lambda_{i+1}}{k(N{\left(1-a\zeta \right)}^{2}-2a\lambda_{i+1}(1-a\zeta )\rho )}$, the wholesale and retail prices of innovative products are positively correlated with *ζ*, the coefficient of consumer purchase regret. When the proportion of new products in the market exceeds $\frac{1}{2}+\frac{aj\rho \lambda_{i+1}}{k(N{\left(1-a\zeta \right)}^{2}-2a\lambda_{i+1}(1-a\zeta )\rho )}$, the wholesale and retail prices of innovative products are negatively correlated with *ζ*, the coefficient of consumer purchase regret.

**Proof**: From Proposition 1, $w_{i}=c_{m}-\frac{1-k(1-2x_{i+1})(1-\zeta )-j}{\rho }e^{-2+\frac{2\rho }{N(1-a\zeta )}\lambda_{i+2}-c\rho }$,$p_{i}=c-\frac{2-2(k(1-2x_{i+1})(1-\zeta )-j)}{\rho }e^{-2+\frac{2\rho }{N(1-a\zeta )}\lambda_{i+2}-c\rho }$. The first-order partial derivatives of *ζ* for *w*_*i*_ and *p*_*i*_, respectively, yields $\frac{\partial w_{i}}{\partial \zeta }=\frac{2aj\rho \lambda_{i+2}+k(1-2x_{i+1})(N{\left(1-a\zeta \right)}^{2}-2a\lambda_{i+2}(1-\zeta )\rho )}{N{\left(1-a\zeta \right)}^{2}\rho }e^{-2-c\rho +\frac{2\rho \lambda_{i+2}}{N-aN\zeta }}$, $\frac{\partial p_{i}}{\partial \zeta }=\frac{4aj\rho \lambda_{i+2}+2k(1-2x_{i+1})(N{\left(1-a\zeta \right)}^{2}-2a\lambda_{i+2}(1-\zeta )\rho )}{N{\left(1-a\zeta \right)}^{2}\rho }e^{-2-c\rho +\frac{2\rho \lambda_{i+2}}{N-aN\zeta }}$. Thus, when $x_{i}<\frac{1}{2}+\frac{aj\rho \lambda_{i+1}}{k(N{\left(1-a\zeta \right)}^{2}-2a\lambda_{i+1}(1-a\zeta )\rho )}$, $\frac{\partial w_{i}}{\partial \zeta }>0$, $\frac{\partial p_{i}}{\partial \zeta }>0$. Conversely, when $x_{i}>\frac{1}{2}+\frac{aj\rho \lambda_{i+1}}{k(N{\left(1-a\zeta \right)}^{2}-2a\lambda_{i+1}(1-a\zeta )\rho )}$, $\frac{\partial w_{i}}{\partial \zeta }<0$, $\frac{\partial p_{i}}{\partial \zeta }<0$.

Corollary 1 and Corollary 2 show that the price strategies of manufacturers and retailers are influenced by external factors in the sale of innovative items, and the effect of customer purchase regret psychology on pricing differs depending on the product’s market share. This means that when the market capacity is certain and consumer regret induces return behavior, it is necessary for manufacturers’ and retailers’ pricing to take into account the innovative product’s share of the market as well as the impact of consumer purchase regret.

**Corollary 3**: When there is no competition between the manufacturer and the retailer and both adopt the optimal strategy for pricing, their sales profits are always equal at all stages and their final sales profits are also equal.

**Proof**: By substituting *w*_*i*_ and *p*_*i*_ into the respective profit functions of the manufacturer and the retailer, it can be obtained that:

$\pi_{M}=\sum_{i=0}^{n-1}\left(\frac{1}{\rho }-\frac{\lambda_{i+1}}{N(1-a\zeta )})N(1-a\zeta )e^{-2+\frac{2\rho }{N(1-a\zeta )}\lambda_{i+1}-c\rho }(j+k(1-\zeta )x_{i})(1-x_{i}\right)$, $\pi_{R}=\sum_{i=0}^{n-1}\left(\frac{1}{\rho }-\frac{\lambda_{i+1}}{N(1-a\zeta )})N(1-a\zeta )e^{-2+\frac{2\rho }{N(1-a\zeta )}\lambda_{i+1}-c\rho }(j+k(1-\zeta )x_{i})(1-x_{i}\right)$. It is obvious that the sales profits are equal in all stages and *π*_*M*_ = *π*_*R*_ holds.

Corollary 3 shows that the optimal wholesale and retail prices of an innovative product can lead to the same maximum profit for the manufacturer and the retailer. This means that when the information between the manufacturer and the retailer is completely open and transparent, and the retailer buys the product exclusively from the manufacturer and sells it directly to the consumer, the supply chain reaches Pareto optimality.

**Corollary 4**: $\frac{\partial D_{n}}{\partial \zeta }<0$, $\frac{\pi_{M}}{\partial \zeta }=\frac{\pi_{R}}{\partial \zeta }<0$, $\frac{\pi_{M}}{\partial m_{e}}=\frac{\pi_{R}}{\partial m_{e}}<0$.

Corollary 4 suggests that the market sales of innovative products decrease as consumer purchase regret *ζ* increases. Moreover, manufacturers’ and retailers’ sales profits decrease as consumer purchase regret increases, and as return rates *m*_*e*_ increase. This means that consumer purchase remorse can impede the spread of innovative products in the market and put the revenues of producers and retailers at risk.

## 5. Numerical analysis

To validate the conclusions in Section 4, this section aims to accomplish the following two points through numerical analysis: (1) comparing and analyzing the optimal pricing of the innovative product, its share of the market, and the manufacturer’s optimal profit at each stage of the sales process; (2) studying the impact of consumer purchase regret psychology on decision variables, sales of innovative products, and profits of members of the supply chain.

The parameter settings in reference [7] are given that the basic parameters in this paper are as shown in Table 3:

**Table 3:** Values of basic parameters in the supply chain.

*ρ*	*c*_*m*_	*c*_*r*_	*k*		*j*	*ζ*	*a*	*n*	*N*
0.003	100	50	0.5	0.8	0.1	0.37		15	100000

Firstly, this paper explores the optimal pricing strategy for each decision maker in the supply chain at each stage, given the value of the consumer purchase regret coefficient. Let *ζ* = 0.1 to get the value of the decision maker’s optimal pricing at each stage, as shown in Table 4:

**Table 4:** Values of basic parameters in the supply chain.

Stage	*w*_*i*_	*p*_*i*_
1	444.19	838.38
2	445.54	841.08
3	446.81	843.63
4	448.02	846.03
5	449.15	848.30
6	450.21	850.42
7	451.21	852.41
8	452.14	854.28
9	453.00	856.00
10	453.86	857.73

As can be seen in Table 4: the wholesale and retail prices of the innovative products increased slowly as the sales process progressed, with an increase of 9.67 in the wholesale price and 19.35 in the retail price. The wholesale and retail prices at any stage satisfy 2*w*_*i*_ − *p*_*i*_ = 50, which is also the difference between the manufacturer’s and retailer’s costs: *c*_*m*_ − *c*_*r*_ = 50.

**Fig 1:**
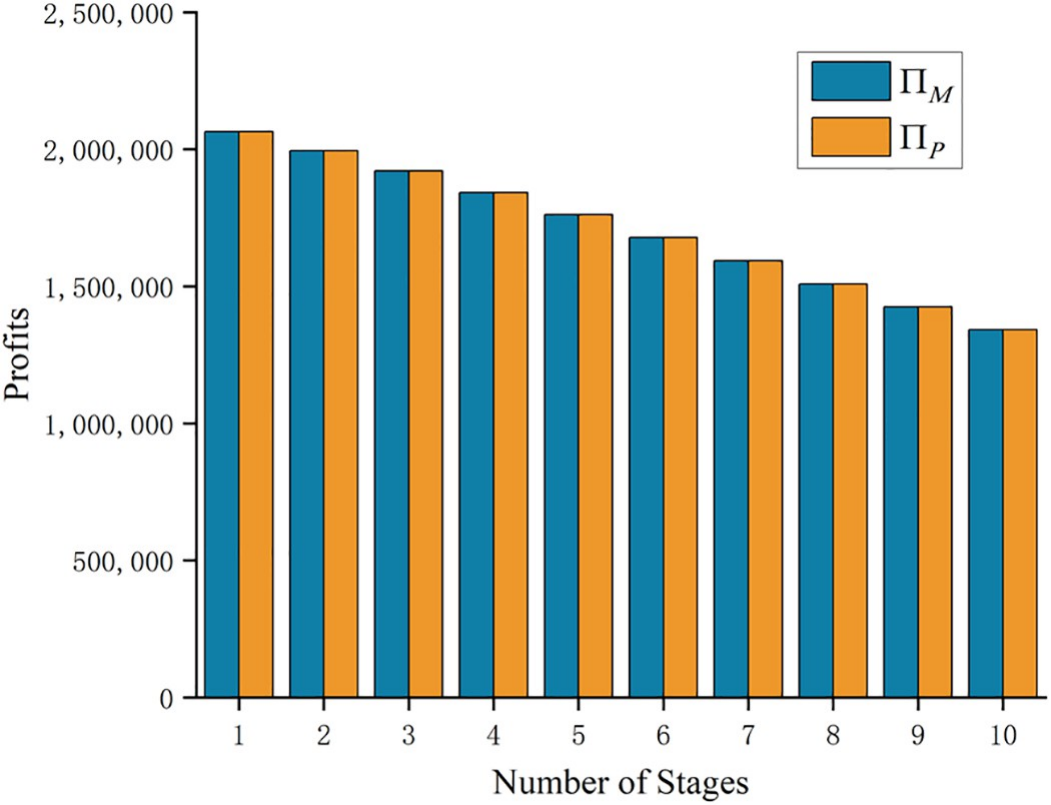
Sales profits for manufacturers and retailers at each stage of the process.

Based on the optimal price of the innovative product obtained from Table 4, the sales profit of the manufacturer and the retailer at each stage is calculated as in Fig 1:

As shown in Fig 1: the sales profit of both the manufacturer and retailer remains constant during each stage, both can reach the maximum at the initial stage when the innovative product enters the market, and as the sales process progresses, the sales profit of both parties eventually diminishes. From the 1st stage to the 10th stage, the sales profit of the manufacturer and retailer decreases by 35.03%. Given that the earnings of both the manufacturer and retailer are the same, we will focus solely on analysing the change in the manufacturer’s profit for the sake of simplicity in further study.

**Fig 2:**
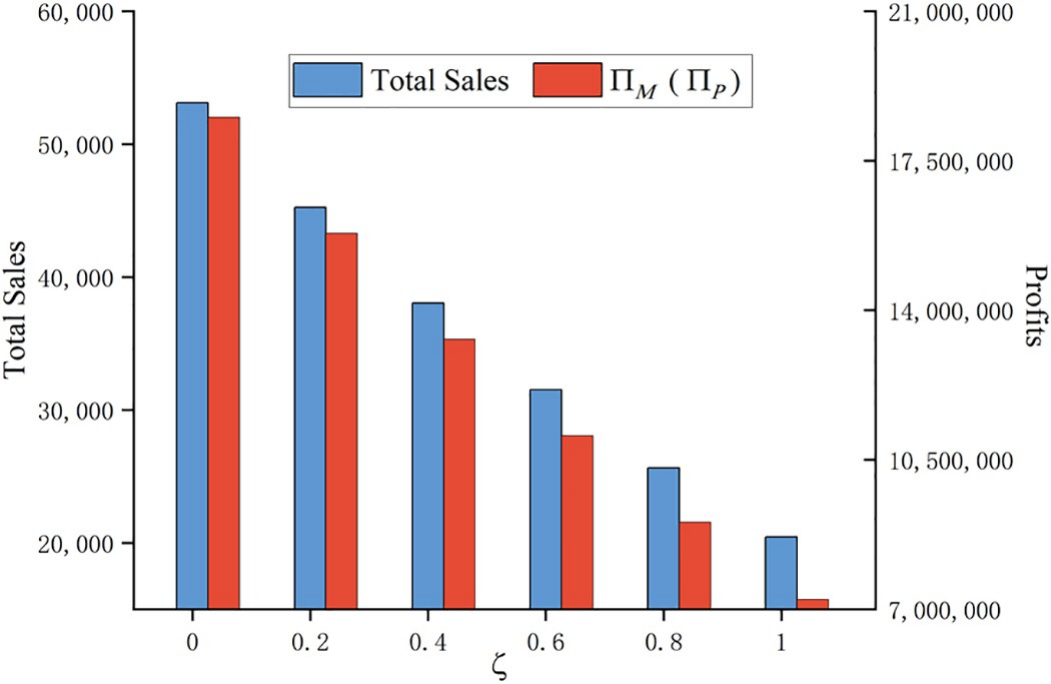
Impact of regret coefficient ζ on actual sales volume and sales profit.

Secondly, this paper considers the consumer purchase regret coefficient *ζ* as a variable and explores its impact on the supply chain. With a step size of 0.2, for *ζ* ∈ (0,1), other parameters remain unchanged, to obtain the actual total sales of the innovative product and the trend of the manufacturer’s (retailer’s) sales profit in the whole sales process, as shown in Fig 2:

As depicted in Fig 2, both actual sales volume and manufacturer’s (retailer’s) sales profits decrease as the coefficient of consumer purchase regret *ζ* increases. When consumers have no purchase regret psychology, i.e., *ζ* = 0, the return rate of the innovative product is 0, both the actual sales volume and the manufacturer’s (retailer’s) sales profit are maximized, at 53,099 and 18,515,378, respectively. When there is a strong consumer purchase regret in the market, i.e., *ζ* = 1, the return rate of the innovative product reaches the maximum, the consumers who have purchased the product have an extreme negative impact on the product, and the actual sales volume and the sales profit of the manufacturer (retailer) are at the minimum, which are 20,452 and 7,233,719, respectively.

**Fig 3:**
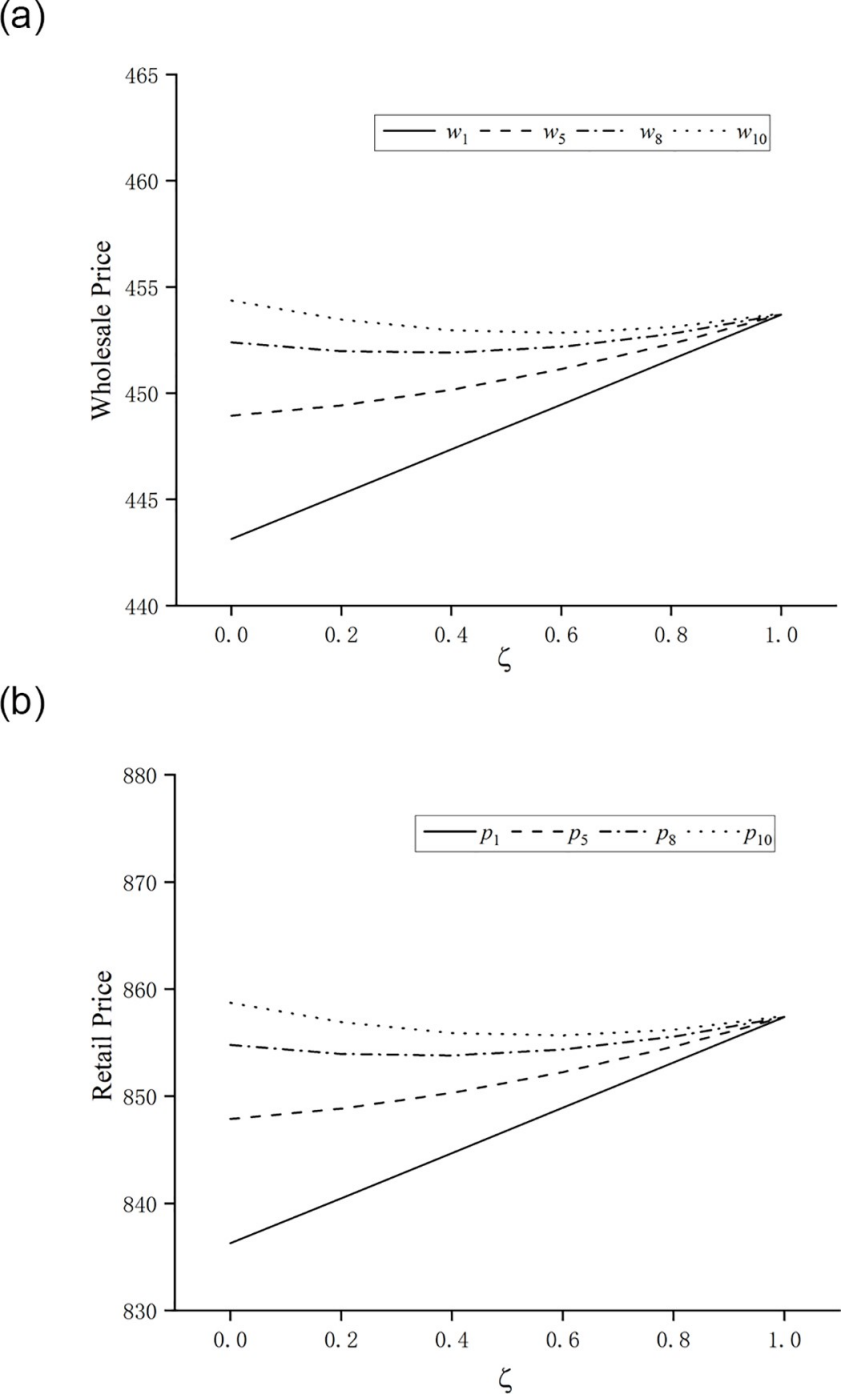
The effect of consumer purchase regret on the wholesale and retail prices of new products. (**a**) Impact of regret coefficient ζ on the wholesale price *w*_*i*_; (**b**) Impact of regret coefficient ζ on the retail price *p*_*i*_.

**Fig 4:**
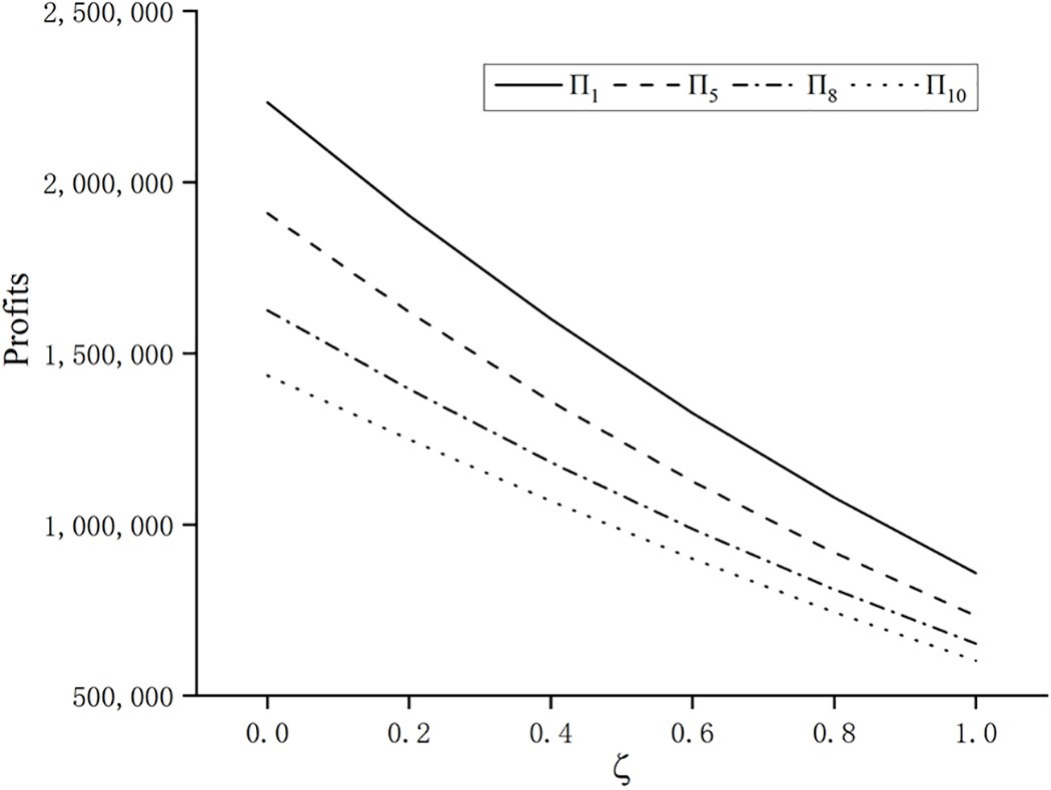
Impact of regret coefficient ζ on manufacturer’s (retailer’s) sales profit.

Thirdly, this paper considers the coefficient of consumer purchase regret *ζ* as a variable and studies the impact of consumer purchase regret on product pricing and profits of manufacturers and retailers at various stages. The four stages of 1, 5, 8 and 10 are selected, in which stages 1 and 5 satisfy the share of innovative products in the market $x_{i}<\frac{1}{2}+\frac{aj\rho \lambda_{i+1}}{k(N{\left(1-a\zeta \right)}^{2}-2a\lambda_{i+1}(1-a\zeta )\rho )}$, in stage 8, when *ζ* = 0 or *ζ* = 0.2, the share of innovative products in the market satisfies $x_{i}>\frac{1}{2}+\frac{aj\rho \lambda_{i+1}}{k(N{\left(1-a\zeta \right)}^{2}-2a\lambda_{i+1}(1-a\zeta )\rho )}$, in stage 10, except for *ζ* = 0.8 and *ζ* = 1, the rest value of *ζ* can satisfy the share of innovative products $x_{i}>\frac{1}{2}+\frac{aj\rho \lambda_{i+1}}{k(N{\left(1-a\zeta \right)}^{2}-2a\lambda_{i+1}(1-a\zeta )\rho )}$. With a step size of 0.2, for *ζ* ∈ (0,1), other parameters remain unchanged, to obtain the wholesale price *w*_*i*_, the retail price *p*_*i*_, and the changes in the sales profit of the manufacturer and the retailer at various stage, as shown in Figs 3 and 4:

Firstly, we analyze the impact of changes in the coefficient of consumer purchase regret on wholesale and retail prices. From Fig 3(a) and 3(b), it can be seen that as consumer purchase regret increases in Stage 1 and Stage 5, $x_{i}<\frac{1}{2}+\frac{aj\rho \lambda_{i+1}}{k(N{\left(1-a\zeta \right)}^{2}-2a\lambda_{i+1}(1-a\zeta )\rho )}$ and the optimal wholesale and retail prices of the innovative product increase. In Stage 8, when *ζ* = 0 or *ζ* = 0.2,$x_{i}>\frac{1}{2}+\frac{aj\rho \lambda_{i+1}}{k(N{\left(1-a\zeta \right)}^{2}-2a\lambda_{i+1}(1-a\zeta )\rho )}$ and the optimal wholesale and retail prices of the innovative product decrease with increasing consumer purchase regret; when *ζ* is greater than 0.4, the share of the innovative product in the market is less than $\frac{1}{2}+\frac{aj\rho \lambda_{i+1}}{k(N{\left(1-a\zeta \right)}^{2}-2a\lambda_{i+1}(1-a\zeta )\rho )}$, in which case the optimal wholesale and retail prices of the innovative product increase with the increase of consumer purchase regret. In Stage 10, when *ζ* is less than 0.8, the optimal wholesale price and retail price of the innovative product decrease with the increase of consumer purchase regret, and when *ζ* = 0.8 or *ζ* = 1, the share of the innovative product in the market is less than $\frac{1}{2}+\frac{aj\rho \lambda_{i+1}}{k(N{\left(1-a\zeta \right)}^{2}-2a\lambda_{i+1}(1-a\zeta )\rho )}$, the optimal wholesale price and retail price of the innovative product increase with the increase of consumers’ purchase regret. Moreover, when *ζ* = 1, the optimal wholesale price and retail price of the innovative product converge to a certain value, the optimal wholesale price converges to 454, and the optimal retail price converges to 857.

Subsequently, the impact of the change in the coefficient of consumer purchase regret *ζ* on the profit of the manufacturer’s (retailer’s) sales is analyzed. As can be seen in Fig 4, the sales profit of the manufacturer (retailer) decreases as the regret psychology increases, and the later the stage in the market, the stronger the purchase regret psychology of the consumer, and the lower the sales profit of the manufacturer (retailer).

## 6. Discussion and conclusion

In this paper, under discrete-time condition, the supply chain of the diffusion of innovative products in the market is taken as the object of study, and the supply chain differential game problem in which consumers have the purchase regret psychology and regret causes the return behavior is studied.

### 6.1. Conclusion

Firstly, the transversal condition has an impact on both the ideal wholesale and retail costs of the innovative product. As the sales process proceeds, the value of the transversal condition steadily diminishes, necessitating the manufacturer and retailer to correspondingly raise the prices of innovative products. Furthermore, there is an equivalence between the optimal pricing of the innovative product and its cost, i.e., the difference between the doubled wholesale price and the retail price is always equal to the difference between the manufacturer’s and the retailer’s costs.

Secondly, the coefficient of consumer purchase regret has a direct impact on the ideal wholesale and retail prices of new items. Furthermore, this impact is closely linked to the market share of innovative products. When the proportion of innovative products in the market is below $\frac{1}{2}+\frac{aj\rho \lambda_{i+1}}{k(N{\left(1-a\zeta \right)}^{2}-2a\lambda_{i+1}(1-a\zeta )\rho )}$, the optimal wholesale and retail prices will gradually increase as consumers develop purchase regret. When the proportion of innovative products in the market exceeds $\frac{1}{2}+\frac{aj\rho \lambda_{i+1}}{k(N{\left(1-a\zeta \right)}^{2}-2a\lambda_{i+1}(1-a\zeta )\rho )}$, the optimal wholesale and retail prices will gradually decrease as consumers develop purchase regret.

Thirdly, both sales of innovative products and sales profits of supply chain members can be affected by consumer purchase regret. As consumer purchase regret increases, the sales volume of the product gradually decreases, and the sales profits of supply chain members gradually decreases. And since there is a positive correlation between the return rate and purchase regret, an increase in purchase regret increases the return rate of innovative products, which further leads to a decrease in the sales volume of the product and the sales profit.

### 6.2. Management insight

Firstly, in the early sales of innovative products into the market, consumers in the freshness of the drive will be more willing to buy the product, the product will have higher sales. At this time, the manufacturer and the retailer need to adopt a lower pricing strategy, in order to be able to quickly promote the innovative products. As sales proceed and the innovative product’s share of the market increases, the product loses its "brand new" concept, which in turn leads to a decline in consumer enthusiasm and sales, thus requiring the manufacturer and the retailer to increase the price in order to increase profits.

Secondly, when the psychology of consumer acquisition regret changes in the market, manufacturers and retailers need to price innovative products according to their share of the market. Specifically, when the share of innovative products in the market is less than $\frac{1}{2}+\frac{aj\rho \lambda_{i+1}}{k(N{\left(1-a\zeta \right)}^{2}-2a\lambda_{i+1}(1-a\zeta )\rho )}$, the wholesale price and the retail price of the product should be gradually increased. This is because when an innovative product enters the market at the initial stage, it needs to recover the cost of research and development as soon as possible, and the negative impact of the regret psychology and the consumer’s return behavior will lead to the reduction of product sales, the manufacturer’s profit is damaged. Therefore, the manufacturer needs to increase the price to reduce the loss of profit, in the face of a strong market regret psychology, the manufacturer’s price increase behavior can establish the brand image. When the share of innovative products in the market is higher than $\frac{1}{2}+\frac{aj\rho \lambda_{i+1}}{k(N{\left(1-a\zeta \right)}^{2}-2a\lambda_{i+1}(1-a\zeta )\rho )}$, the product already has a certain consumer base in the market. The stronger the purchase regret, the greater the impact on the spread of new products in the market, the higher the return rate of the product, so the manufacturer needs to reduce their price, using “thin margins to obtain as much profit as possible” strategy. In addition, manufacturers also need to meet the demand of consumers as much as possible, and improve after-sales service to reduce the negative publicity caused by consumer regrets as well as the impact of behaviors such as returns on total product sales.

Thirdly, compared with the traditional static supply chain, the supply chain of innovative products in discrete time is more relevant to reality, which the dynamic demand for products in the market is more complex, and the formulation of optimal prices for innovative products is more difficult. Therefore, it is important for decision makers in the supply chain to improve their ability to acquire and process market information, and to be able to adjust their strategies in response to market changes.

### 6.3. Future research

In reality, the supply chain has numerous manufacturers and suppliers, and the decision-making relationships between the various vendors in the supply chain are complex. However, this paper solely focuses on a straightforward supply chain and only considers the case where the manufacturer leads and the retailer follows. Therefore, it is feasible to broaden the participants of the supply chain based on this paper by considering the impact of consumer purchase regret on the most advantageous decision-making process of the supply chain when multiple manufacturers or retailers are involved, or when there are complex relationships between multiple manufacturers and retailers, such as competition or cooperation among them, further research could also be conducted on retailer-led supply chain decisions.

## Supporting information

S1 Data (XLSX)
